# Use of coffee husks – comparison of pellet bedding quality, performance features, and some welfare indicators of broiler chickens

**DOI:** 10.1186/s12917-023-03749-3

**Published:** 2023-10-02

**Authors:** Jakub Biesek, Mirosław Banaszak, Sebastian Wlaźlak, Marek Adamski

**Affiliations:** Department of Animal Breeding and Nutrition, Faculty of Animal Breeding and Biology, Bydgoszcz University of Science and Technology, Mazowiecka 28, 85-084 Bydgoszcz, Poland

**Keywords:** Bedding chemical composition, Coffee waste, Efficiency production, Litter quality, Poultry manure, Skin lesions

## Abstract

**Background:**

The study aimed to evaluate the influence of wheat straw and different coffee husk (CHs) levels in pellet bedding on its quality, broiler chickens’ performance, meat quality, and welfare indicators. In total, 200 Ross 308 chickens were divided into 4 groups: C – control with wheat straw pellet; CH10 – pellet with 10% CHs, CH25 – pellet with 25% CHs, and CH50 – pellet with 50% CHs. During 42 days of rearing, each bedding's physicochemical features were analyzed. The production results were controlled, and the footpad dermatitis, hock burns, and feather quality were assessed. From chosen birds, carcass composition was analyzed, as well as the qualitative features (color, water-holding capacity, drip loss) and breaking bone strength.

**Results:**

The bedding material and rearing days influenced the content of dry matter, crude fiber, nitrogen, phosphorus, potassium, NDF, ADF, and pH. The results were inconclusive. The increasing trends in nitrogen, phosphorus, and potassium content were noticed at the end of rearing. Strong coefficient determination in bedding features was found (0.580 – 0.986). The pellet with CHs had no adverse effect on the growth performance of broilers. In the CH50 group, a lower fat percentage was found. A beneficial effect on water-holding capacity was noticed in leg muscles from CH10 and pectoral muscles from CH25. A significant decrease was found in footpad dermatitis incidence in groups CH25 and CH50.

**Conclusions:**

It can be concluded that CHs reuse in broilers as the pellet bedding material is possible due to the beneficial effect on some meat quality features and no adverse effect on the performance of broiler chickens. The positive impact on lower foot pad dermatitis incidence indicated the possibility of using CHs in pellet bedding.

## Background

Coffee production is recognized worldwide, with an annual value of $ 200 billion, assuming 10.5 million tons of coffee beans. Roasted coffee is produced from coffee beans, a product destined for the consumer market [1]. The largest coffee exporters are Brazil, Colombia, Vietnam, and Indonesia [2]. Poland is one of the leaders (10^th^) among coffee market customers, and the import of coffee grains amounted to 160 thousand tons [3]. By-products (coffee husks (CHs) and pulp) are produced in coffee roasters, and combustion may pollute the environment [4, 5]. CHs are the subject of research on the possibility of their use as fuel, alternative energy, material for producing biodiesel, biogas, biomass briquette, and even fermentable sugars and fertilizer [2, 6–9]. Using CHs as bedding material can be a solution to manage waste and counteract environmental pollution [10].

The CHs might also be used in animal production [11]. As indicated by the cited authors, the commonly used materials for bedding are sawdust, rice husks, sugarcane pulp, sugarcane bagasse, chopped straw, paper mill by-products, sand, and wood shavings, corn cobs, oat hulls, dried leaves, CHs. Ortiz et al. [12, 13] concluded that CHs as bedding (non-pelleted) affected the production results of broiler chickens. Bedding should be cheap, available, non-toxic, and usable as a fertilizer, and its quality (adequate moisture, physical structure) affects the growth, quality of the meat, and the condition of foot pads [14]. Pelleted bedding is often used in poultry production since it ensures higher floor hygiene, is beneficial for poultry condition, and improves growth performance [15, 16]. Lower footpad lesions were noticed when the pellet was used compared to the chopped straw bedding [15]. Footpad dermatitis, one of the welfare indicators, is one of the most common diseases of foot pads, affecting the health status of poultry. It is manifested primarily by hyperkeratosis, scaly lesions, abrasions, ulcerations, swelling, cracks, bleeding, inflammation, and tissue necrosis in the foot pads [17]. Depending on the material used, the bedding may change its physicochemical properties during the rearing of broiler chickens, which is crucial for further reuse [18].

The CHs contain 7 to 18% moisture, and in 100 g of dry matter, 9.5 g of protein, 1.75 g of lipids, 5 g of minerals, and 71.5 g of carbohydrates, on average. It is also a rich source of potassium and other minerals [5]. CHs mixed with manure were reported to enrich the fertilizer [3, 9, 10]. Using poultry manure as a fertilizer can impact its quality. A good-quality fertilizer that enriches the soil should contain an appropriate level of nitrogen and other nutrients and have suitable physicochemical properties, including the pH value related to soil acidification [19].

Thus, using CHs from local coffee roasters as bedding material for broiler chickens may have potential. Poultry manure is a good fertilizer and indicates an increased possibility of using waste. Following pro-environmental activities it aligns with the current poultry production trends and challenges. Using waste coffee by-products might increase the poultry producer's competitiveness and the producer of pellet bedding. Eser et al. [20] concluded that reusing waste products as bedding is essential for the environment.

The study aimed to evaluate and compare the physicochemical qualities of pellet bedding, growth performance, carcass features, meat quality, footpad dermatitis, hock burns, and feather scoring as welfare indicators of broiler chickens kept on pellet bedding with the use of CHs at different levels.

## Results

The coffee husks (CHs) used in our experiment were characterized by dry matter at the level of 93.98 (± 0.32, within the material weight), crude fiber content—29.85 (± 0.37), and pH value—6.46 (± 0.08).

When analyzing the interaction of bedding material and rearing days (Table 1), the highest dry matter was found in the CH50 group on day 14 compared to the C and CH10 groups on day 21 and all from day 28 to day 42 (*P* = 0.001). The highest crude fiber content was found in group C on day 1 (fresh wheat straw pellet), and the lowest values were found on day 42 (*P* < 0.001). Significantly higher nitrogen content in the CH50 group on day 1 was noticed compared to all groups on every rearing day. Since day 7, the nitrogen content was lowered, and on day 35 increased (*P* < 0.001). Phosphorus content decreased, and the potassium content increased within rearing days. However, the highest phosphorus content was found on day 42 in the C and CH10 groups and the lowest in all groups on days 1 and 7 (*P* < 0.001). Potassium content in group CH50 on day 42 was the highest, and the lowest was in group C on day 1 (*P* < 0.001). Simultaneously, the highest NDF and ADF content was noticed in the C group on day 1. The lowest values were found on day 21 in group C (NDF) and for ADF – in CH10 and CH25 groups on day 21 (*P* < 0.001). When comparing pH values, the highest in group CH10 on day 7 and the lowest in the CH10 group on day 28 (*P* < 0.001) were found. A high coefficient of determination in the chemical composition of the bedding for the discussed features (adj. R^2^) was demonstrated, ranging from 0.863—0.986. The value of adj. R^2^ for the pH value was medium–high—0.580.

**Table 1:** Chemical composition and pH value of pellet bedding

Days	Groups^1^	Dry matter	Crude fiber	Nitrogen	Phosphorus	Potassium	NDF^2^	ADF^3^	pH
1	C	82.92^ab^	42.71^a^	2.49^i^	0.07^j^	0.38^p^	75.96^a^	46.74^a^	6.44^bcd^
	CH10	80.53^abc^	40.99^ab^	4.17^c^	0.06^j^	0.73^ mn^	72.15^b^	44.06^bc^	6.60^ab^
	CH25	81.98^abc^	40.00^bc^	6.32^b^	0.06^j^	0.91^ lm^	71.27^b^	44.36^b^	6.58^ab^
	CH50	81.96^abc^	36.54^de^	9.10^a^	0.06^j^	1.10^jk^	63.22^fgh^	42.75^ cd^	6.60^ab^
7	C	77.04^abcd^	42.29^ab^	2.60^i^	0.09^j^	0.44^op^	67.93^ cd^	41.01^efg^	6.24^ef^
	CH10	77.27^abc^	41.81^ab^	4.17^c^	0.09^j^	0.61^no^	69.10^c^	42.43^de^	6.70^a^
	CH25	78.83^abc^	40.53^ab^	4.03^ cd^	0.10^ij^	0.80^ m^	65.30^ef^	42.46^de^	6.60^ab^
	CH50	77.42^abc^	36.32^de^	4.02^ cd^	0.09^j^	1.10^jk^	66.90^de^	41.34^defg^	6.59^ab^
14	C	77.20^abc^	36.45^de^	1.35^ m^	0.20^gh^	0.68^ mn^	61.64^ h^	40.98^efg^	6.24^ef^
	CH10	81.53^abc^	37.70^ cd^	1.38^ m^	0.18^gh^	0.72^ mn^	62.04^gh^	40.71^ fg^	6.35^cdef^
	CH25	83.82^a^	35.81^de^	1.69^ l^	0.18^gh^	0.93^kl^	64.04^ fg^	40.83^ fg^	6.45^bcd^
	CH50	82.97^ab^	34.59^e^	1.98^kl^	0.16^hi^	1.06^jkl^	67.31^cde^	38.47^hi^	6.35^cdef^
21	C	74.19^cdef^	30.27^f^	1.81^ l^	0.30^e^	1.07^jkl^	62.86^gh^	36.38^jk^	6.30^def^
	CH10	75.24^bcde^	30.17^f^	2.18^jk^	0.29^ef^	1.18^ij^	57.26^ij^	34.84^ k^	6.40^cde^
	CH25	78.55^abc^	31.99^f^	2.00^kl^	0.23^ fg^	1.19^ij^	58.67^i^	35.34^ k^	6.36^cde^
	CH50	79.35^abc^	31.31^f^	2.17^jk^	0.21^gh^	1.35^hi^	57.19^ij^	37.75^hij^	6.49^bcd^
28	C	60.32^ h^	25.99^gh^	2.73^hi^	0.43^d^	1.65^efg^	54.45^ k^	36.97^ij^	6.39^cde^
	CH10	67.32^fgh^	26.33^ g^	2.47^ij^	0.35^e^	1.50^gh^	55.36^jk^	37.17^ij^	6.19^f^
	CH25	67.57^efgh^	30.40^f^	2.62^i^	0.34^e^	1.54^ fg^	56.76^ij^	37.25^ij^	6.33^def^
	CH50	69.25^defg^	29.93^f^	2.68^i^	0.33^e^	1.70^def^	57.75^i^	38.97^ h^	6.46^bcd^
35	C	64.63^gh^	23.23^ij^	3.33^ fg^	0.56^b^	2.00^bc^	61.35^ h^	41.25^defg^	6.54^ab^
	CH10	62.50^gh^	24.75^ghi^	3.40^f^	0.49^ cd^	1.85^ cd^	62.77^gh^	41.40^def^	6.51^bc^
	CH25	68.64^efg^	24.25^ghi^	3.02^gh^	0.44^d^	1.73^de^	63.03^gh^	40.66^ fg^	6.52^b^
	CH50	62.31^gh^	23.75^hij^	3.23^ g^	0.47^d^	1.84^ cd^	62.16^gh^	41.31^defg^	6.47^bcd^
42	C	40.60^j^	19.53^ k^	3.80^de^	0.64^a^	2.07^ab^	62.94^gh^	41.13^efg^	6.38^cde^
	CH10	40.27^j^	19.72^ k^	3.58^ef^	0.63^a^	2.08^ab^	64.15^ fg^	41.85^def^	6.48^bcd^
	CH25	50.40^i^	21.79^jk^	3.37^ fg^	0.55^bc^	1.95^bc^	62.46^gh^	40.29^gh^	6.43^bcd^
	CH50	44.09^ij^	20.67^ k^	3.55^ef^	0.59^ab^	2.20^a^	62.68^gh^	41.09^efg^	6.47^bcd^
SEM^4^		0.81	0.45	0.09	0.01	0.03	0.31	0.17	0.01
*P*-value interaction: B × D		0.001	< 0.001	< 0.001	< 0.001	< 0.001	< 0.001	< 0.001	< 0.001
Adj. R^2^, ^5^		0.863	0.962	0.986	0.956	0.961	0.938	0.894	0.580
	C	68.13	31.49	2.59^c^	0.33	1.19^b^	63.87	40.64	6.36^b^
	CH10	69.24	31.64	3.05^bc^	0.30	1.24^b^	63.26	40.35	6.46^a^
	CH25	72.83	32.11	3.29^ab^	0.27	1.29^ab^	63.08	40.17	6.47^a^
	CH50	71.05	30.44	3.82^a^	0.27	1.48^a^	62.46	40.24	6.49^a^
*P*-value bedding material (B)		< 0.001	< 0.001	< 0.001	< 0.001	< 0.001	< 0.001	0.020	< 0.001
1		81.84^a^	40.06^a^	5.52^a^	0.06^f^	0.78^e^	70.65^a^	44.48^a^	6.55^a^
7		77.64^b^	40.24^a^	3.71^b^	0.09^f^	0.74^e^	67.31^b^	41.81^b^	6.53^a^
14		81.38^a^	36.14^b^	1.60^d^	0.18^e^	0.85^e^	63.76^c^	40.25^c^	6.35^d^
21		76.84^b^	30.93^c^	2.04^d^	0.26^d^	1.20^d^	58.99^d^	36.07^e^	6.39^ cd^
28		66.12^c^	28.16^d^	2.63^ cd^	0.36^c^	1.60^c^	56.08^e^	37.59^d^	6.34^d^
35		64.52^c^	23.99^e^	3.25^bc^	0.49^b^	1.85^b^	62.33^c^	41.15^b^	6.51^ab^
42		43.84^d^	20.43^f^	3.57^b^	0.60^a^	2.08^a^	63.06^c^	41.09^bc^	6.44^bc^
*P*-value days of rearing (D)		< 0.001	< 0.001	< 0.001	< 0.001	< 0.001	< 0.001	< 0.001	< 0.001

^a,^^b…,^ values marked with different letters differ within groups (bedding material or days), with *P*-value < 0.05

^1^C, wheat straw pellet; CH10, pellet with 90% of wheat straw and 10% of coffee husks; CH25, pellet with 75% of wheat straw and 25% of coffee husks; CH50, pellet with 50% of wheat straw and 50% of coffee husks

^2^NDF, neutral detergent fiber

^3^*ADF* acid detergent fiber

^4^*SEM* standard error of the mean

^5^*adj*. *R*^2^ adjustable coefficient of determination

Considering the bedding material effect, the highest nitrogen content was found in the CH50 group and the lowest in the C group (*P* < 0.001). In turn, the potassium content was lower in the C and CH10 groups than in the CH50 group (*P* < 0.001). In all groups where CHs pellets were used, the pH was higher than in the control group (*P* < 0.001) (Table 1).

During rearing, the dry matter and the crude fiber content decreased (*P* < 0.001). Nitrogen content decreased until day 21 and increased from day 35 (*P* < 0.001). An increasing trend in phosphorus content was found (*P* < 0.001), similarly to potassium (*P* < 0.001). The content of NDF and ADF decreased each week of rearing, and from days 28—35, there was an increase (*P* < 0.001). Also, the pH value of the bedding material (without division into its type) decreased on day 14 and increased on day 35 (*P* < 0.001) (Table 1).

The physical properties of freshly prepared pellet bedding made of wheat straw or with the share of CHs at 10, 25, and 50% are presented in Table 2. No statistically significant differences were found in the pellet cutting strength (*P* = 0.256, 0.316). The water absorption capacity of all the proposed pellets was approximately five times higher than the initial weight of the bedding. By measuring the bedding moisture content on a 5-point scale (subjective assessment), it was shown that the bedding quality (higher moisture content) was statistically significantly worse in the control group (3.10 points) than in the groups kept on a pellet with CHs (1.00 – 1.60 points) (*P* < 0.001).

**Table 2:** Physical features of freshly prepared pellet bedding and used litter scale

Item^1^	Group^2^				SEM^3^	*P*-value
	C	CH10	CH25	CH50		
Firmness (N)	75.96	105.61	90.86	96.78	5.23	0.256
Toughness (N*sec)	45.63	70.47	64.98	43.00	5.95	0.316
Absorptive capacity (%) 24 h	523.43	521.89	522.69	515.43	5.32	0.958
Absorptive capacity (%) 48 h	496.83	486.00	515.00	496.83	4.40	0.129
Litter assessment scale (points)	3.10^a^	1.60^b^	1.00^b^	1.30^b^	0.20	< 0.001

^1^Litter assessment scale, 5-points scale modified from Welfare Quality Assessment Protocol (Welfare Quality R Consortium, 2009)

^2^Ccontrol group kept on the wheat straw bedding; CH10, group held on the bedding with 10% of coffee husks and 90% of wheat straw; CH25, group kept on the bedding with 25% of coffee husks and 75% of wheat straw; CH50, group held on the bedding with 50% of coffee husks and 50% of wheat straw

^3^*SEM* standard error of the mean

In Table 3, the production results of broiler chickens are presented. At the beginning of rearing, deaths from 8 to 12% of birds were recorded in groups. However, it was related to the poor condition status of the chicks in the first week of rearing. The body weight and feed intake indicators were similar in all of groups. Only in the fourth week of rearing in the CH10 group, where chickens were kept on a pellet with a 10% share of CHs, the growth rate was significantly lower than in the CH50 group, where chickens were kept on a pellet with a 50% share of CHs (*P* = 0.019).

**Table 3:** Growth performance of broiler chickens

Item^1^		Group^2^				SEM^3^	*P*-value
		C	CH10	CH25	CH50		
Viability (%)		100	92	88	92	2.19	0.277
BW (g)	Day 1	38.22	38.76	38.22	37.82	0.17	0.289
	Day 7	122.88	114.03	105.00	104.18	4.46	0.434
	Day 14	354.81	326.73	313.09	306.18	11.58	0.492
	Day 21	743.26	721.67	713.32	685.79	14.50	0.604
	Day 28	1318.44	1254.16	1280.10	1267.96	25.23	0.850
	Day 35	1921.68	1869.00	1852.28	1839.57	35.11	0.873
	Day 42	2543.98	2453.70	2429.28	2404.78	42.40	0.707
The growth rate (%)	Week 1	104.99	95.99	89.05	93.11	3.66	0.495
	Week 2	97.13	96.57	100.35	98.38	1.38	0.803
	Week 3	70.75	76.10	79.31	76.68	1.94	0.494
	Week 4	55.78^ab^	53.63^b^	57.03^ab^	59.56^a^	0.76	0.029
	Week 5	37.25	39.57	36.66	36.58	0.81	0.554
	Week 6	27.89	27.38	26.80	26.65	1.07	0.980
ADBWG (g)	Days 1–14	22.61	20.57	19.63	19.17	0.82	0.492
	Days 15–35	74.61	73.44	73.29	73.02	1.32	0.061
	Days 36–42	88.90	83.53	82.43	80.74	3.35	0.864
	Total	59.66	57.50	56.93	56.36	1.01	0.707
ADFI (g)	Days 1–14	35.24	33.34	32.33	31.95	0.83	0.539
	Days 15–35	127.51	126.98	124.81	127.54	1.12	0.828
	Days 36–42	150.70	153.22	148.48	148.04	1.27	0.480
	Total	100.62	100.38	99.80	99.35	0.77	0.118
FCR (kg/kg)	Days 1–14	1.56	1.65	1.70	1.68	0.04	0.606
	Days 15–35	1.71	1.75	1.70	1.76	0.03	0.896
	Days 36–42	1.71	1.84	2.06	1.84	0.11	0.722
	Total	1.69	1.76	1.77	1.77	0.03	0.816
EPEF		359.22	315.63	292.23	303.07	14.27	0.388
EBI		353.82	310.72	287.65	298.33	14.10	0.389

^a,^^b^^…,^ different letters in the row show statistically significant differences between the groups, *P*-value < 0.05

^1^*BW* body weight, *ADBWG* average daily body weight gain, *ADFI* average daily feed intake, *FCR* feed conversion ratio, *EPEF* European Production Efficiency Factor, *EBI* European Broiler Index

^2^*C* control group kept on the wheat straw bedding, CH10, group held on the bedding with 10% of coffee husks and 90% of wheat straw, CH25, group kept on the bedding with 25% of coffee husks and 75% of wheat straw, CH50, group held on the bedding with 50% of coffee husks and 50% of wheat straw

^3^standard error of the mean

Carcass weight with offal was significantly lower in the CH25 (*P* = 0.004) and CH50 (*P* = 0.015) groups than in the C group, as well as the pre-slaughter body weight (*P* = 0.004; 0.025, respectively) and carcass weight (*P* = 0.004; 0.018, respectively). The heart weight in the CH25 group was significantly lower than in the C group (*P* = 0.004), as was the weight of the pectoral muscles (*P* = 0.034). In the CH50 group, the weight of skin with subcutaneous fat in the carcass was significantly lower than in the other groups (*P* = 0.001). The weight of total fatness was lower in the CH50 group than in the C group (*P* = 0.018). In group C, a significantly higher carcass remains weight was demonstrated compared to groups C25 (*P* = 0.015) and C50 (*P* = 0.029) (Table 4).

**Table 4:** Weight of the broiler chicken carcass and its elements

Item (g)	Group^1^				SEM^2^	*P*-value
	C	CH10	CH25	CH50		
Pre-slaughter body weight	2721.50^a^	2539.10^ab^	2464.90^b^	2515.90^b^	28.09	0.004
Carcass weight	2002.59^a^	1862.40^ab^	1784.66^b^	1818.20^b^	24.03	0.004
Carcass with offal weight	2094.25^a^	1952.15^ab^	1875.02^b^	1907.20^b^	24.01	0.003
Heart	15.47^a^	13.33^ab^	12.09^b^	13.84^ab^	0.36	0.006
Liver	51.79	48.86	51.59	49.40	1.38	0.842
Gizzard	24.40	27.56	26.68	25.76	1.07	0.766
Neck	64.36	62.70	59.97	60.17	1.59	0.740
Pectoral muscle	609.34^a^	552.60^ab^	523.76^b^	581.43^ab^	11.40	0.042
Leg muscle	392.97	394.55	367.96	355.01	7.12	0.133
Total muscle	1002.31	947.15	891.72	936.44	15.86	0.099
Skin with subcutaneous fat	183.35^a^	168.36^a^	168.63^a^	140.96^b^	4.05	0.001
Abdominal fat	15.71	18.89	17.55	22.91	1.12	0.129
Fatness	199.06^a^	187.25^ab^	186.18^ab^	163.87^b^	4.35	0.029
Wings with skin	182.38	179.66	175.62	179.61	2.58	0.843
Carcass remains	554.48^a^	485.64^ab^	471.17^b^	478.11^b^	10.36	0.010

^a,^^b…,^ different letters in the row show statistically significant differences between the groups, *P*-value < 0.05

^1^*C* control group kept on the wheat straw bedding, CH10 group held on the bedding with 10% of coffee husks and 90% of wheat straw, CH25 group held on the bedding with 25% of coffee husks and 75% of wheat straw, CH50 group held on the bedding with 50% of coffee husks and 50% of wheat straw

^2^*SEM* standard error of the mean

However, the carcass yield was similar in all groups (72.20–73.57%, *P* = 0.219). The percentage of skin with subcutaneous fat in the carcass was lower in the CH50 group compared to the others (*P* = 0.001). On the other hand, the percentage of abdominal fat in the CH50 group was significantly higher than in the C group (*P* = 0.011). The share of fatness in the carcass in the CH50 group was significantly lower than in the CH25 group (*P* = 0.026) (Table 5).

**Table 5:** Slaughter yield of broiler chicken and carcass elements percentage

Item (%)	Group^1^				SEM^2^	*P*-value
	C	CH10	CH25	CH50		
Slaughter yield	73.57	73.34	72.40	72.20	0.28	0.219
Slaughter yield with offal	76.93	76.90	76.06	75.76	0.25	0.233
Heart	0.78	0.72	0.68	0.76	0.02	0.222
Liver	2.58	2.64	2.90	2.74	0.08	0.533
Gizzard	1.22	1.50	1.50	1.45	0.07	0.395
Neck	3.22	3.37	3.36	3.30	0.08	0.904
Pectoral muscle	30.46	29.69	29.31	31.82	0.37	0.072
Leg muscle	30.23	29.96	29.76	28.93	0.99	0.973
Total muscle	50.06	50.87	49.94	51.33	0.41	0.593
Skin with subcutaneous fat	9.13^a^	9.05^a^	9.44^a^	7.78^b^	0.17	0.001
Abdominal fat	0.77^b^	1.01^ab^	0.98^ab^	1.25^a^	0.06	0.021
Fatness	9.90^ab^	10.06^ab^	10.42^a^	9.03^b^	0.18	0.036
Wings with skin	9.14	9.64	9.84	9.89	0.12	0.121
Carcass remains	27.69	26.06	26.44	26.45	0.45	0.611

^a,^^b…,^ different letters in the row show statistically significant differences between the groups, *P*-value < 0.05

^1^*C* control group kept on the wheat straw bedding; CH10 group held on the bedding with 10% of coffee husks and 90% of wheat straw, CH25 group held on the bedding with 25% of coffee husks and 75% of wheat straw, CH50 group held on the bedding with 50% of coffee husks and 50% of wheat straw; ^2^SEM, standard error of the mean

In Table 6, the qualitative physicochemical characteristics of broiler chickens' pectoral and leg muscles are presented. Muscle acidification (pH) was similar in all groups (5.88–5.91). Pectoral muscles from the CH25 group showed a significantly lower water-holding capacity value (WHC) than in the CH10 group (*P* = 0.016). In leg muscles, a significantly higher yellowness (b*) value was demonstrated in the CH10 group compared to the C group (*P* = 0.017). In the CH25 group, the highest WHC of leg muscle was found compared to group C (*P* = 0.033), group CH10 (*P* < 0.001), and group CH50 (*P* < 0.001). At the same time, the CH10 group was characterized by the lowest WHC in leg muscle compared to groups C (*P* = 0.033) and CH25 (*P* < 0.001).

**Table 6:** Physicochemical features of broiler chicken’s muscles and the breaking strength of the leg bones

Item^1^		Group^2^				SEM^3^	*P*-value
		C	CH10	CH25	CH50		
Pectoral muscle	pH_24_	5.90	5.88	5.91	5.88	0.01	0.707
	L*—color	52.08	52.46	52.20	50.32	0.50	0.428
	a*—color	2.53	2.68	2.81	2.79	0.13	0.870
	b*—color	4.63	4.40	4.70	4.12	0.26	0.864
	Drip loss (%)	1.52	1.58	1.73	1.38	0.16	0.900
	WHC (%)	35.05^ab^	37.45^a^	32.52^b^	35.62^ab^	0.60	0.028
Leg muscle	L*—color	50.50	51.99	50.58	49.57	0.44	0.286
	a*—color	7.03	8.90	9.43	8.85	0.39	0.147
	b*—color	4.40^b^	6.66^a^	5.26^ab^	5.22^ab^	0.28	0.027
	WHC (%)	35.60^b^	33.04^c^	40.69^a^	34.38^bc^	0.55	< 0.001
Breaking strength of tibia bone (N)		340.52	284.04	276.17	338.33	14.36	0.231
Breaking strength of femur bone (N)		200.71	210.34	211.92	240.36	10.55	0.588

^a,^^b^^…,^ different letters in the row show statistically significant differences between the groups, *P*-value < 0.05

^1^L*—lightness; a*—redness; b*—yellowness, *WHC* water-holding capacity

^2^*C* control group kept on the wheat straw bedding, CH10 group held on the bedding with 10% of coffee husks and 90% of wheat straw, CH25 group held on the bedding with 25% of coffee husks and 75% of wheat straw, CH50 group held on the bedding with 50% of coffee husks and 50% of wheat straw

^3^*SEM* standard error of the mean

Footpad dermatitis (FPD) was assessed following the point scale (Table 7). The significantly highest share of chickens in the CH25 and CH50 groups with a score of 0 (95.78–98%), and no lesions were found, compared to the control group (58%). On the other hand, significantly, the highest share of broilers with tiny skin lesions as well as discoloration and superficial changes indicative of hyperkeratosis (score 1) was found in group C (42%), compared to groups C25 and C50, where the share of birds with FPD was 2.00—4.22%. Similarly, significant differences were found on the point scale (*P* = 0.024). Statistical analysis of hock burns and feathers scoring showed no significant differences between the groups (*P* > 0.05). Despite the lack of statistical confirmation, a quantitative trend favored a higher share of CHs in the pellet bedding. The table does not show FPD score 2 and hock burns score 3; as such, results were not noticed.

**Table 7:** Incidence and footpad dermatitis score, hock burns, and the feather quality

Item^1^ (% in groups)	Group^2^				SEM^3^	*P*-value
	C	CH10	CH25	CH50		
FPD score 0	58.00^b^	87.78^ab^	98.00^a^	95.78^a^	5.44	0.024
FPD score 1	42.00^a^	12.22^ab^	2.00^b^	4.22^b^	5.54	0.024
FPD score, points	21.00^a^	6.11^ab^	1.00^b^	2.11^b^	2.77	0.024
Hock burns score 1	66.00	77.33	81.70	73.06	3.36	0.424
Hock burns score 2	34.00	22.67	18.30	26.94	3.36	0.424
Feather score 1	0.00	0.00	2.22	4.22	0.88	0.275
Feather score 2	46.00	47.37	55.40	64.06	4.17	0.418
Feather score 3	54.00	50.41	42.38	31.72	4.35	0.288

^a,^^b…,^ different letters in the row show statistically significant differences between the groups, *P*-value < 0.05

^1^FPD score: 0, indicated no or very small superficial changes, slight discoloration of the skin of the sole, and mild hyperkeratosis; 1, indicated mild skin changes and discoloration, as well as superficial changes, dark warts, and hyperkeratosis; 2; indicated severe lesions, ulcerations, and crusts, as well as signs of hemorrhages and swollen soles of the paws; Hock burns score: 1, no burns; 2, moderate burns; 3, severe burns; Feather score: 1, no visible skin, full feather coverage; 2, relatively small area of skin exposed; 3, rather large skin exposure area

^2^*C* control group kept on the wheat straw bedding, CH10 group held on the bedding with 10% of coffee husks and 90% of wheat straw, CH25 group held on the bedding with 25% of coffee husks and 75% of wheat straw, CH50 group held on the bedding with 50% of coffee husks and 50% of wheat straw

^3^*SEM* standard error of the mean

## Discussion

Živkov Baloš et al. [21] analyzed the chemical composition of various bedding materials (cellulose pellets, wood chips, peat, and pH stabilizers). The authors showed moisture, pH, nitrogen, potassium, and phosphorus content changes over 5 weeks. An increase in humidity was also demonstrated. The content of nitrogen, potassium, and phosphorus increased with the passage of successive days of broiler rearing, as in our research. It may indicate a high nitrogen content of wheat straw and CHs, shown in fresh bedding material. The decrease in the content of substances (including nitrogen) may be related to their natural release, and the increase in the following days was caused by chicken manure [22, 23]. The fiber content in bedding material (wheat straw, clover straw, cornstalk chip, sugarcane top chips, chopped palm spines, corn ear husks) in the study by Farghly et al. [14] was in the range of 20.30—40.30%. Similar results were obtained in our study (20.43—40.24%). The proportion of lignin causes a higher proportion of fiber as the main component of plant tissue [24]. Adding the CHs may have reduced the lignin level in the litter. Higher fiber content facilitates digestion by chickens [24]. A similar mechanism can be found in the distribution of manure. The lower fiber content (lignin) could be related to the faster degradation of the used bedding material [25]. As described by the authors [21], the nitrogen content is related to ammonia, and its higher share in used litter indicates its higher accumulation and abundance. It suggests the possibility of using manure as fertilizer. Differences between the content of particular components may result from using different bedding materials, rearing technology, poultry houses' hygiene, as well as the protein content of the feed. Garces et al. [26] investigated the quality of various types of bedding material (sand, coconut husk, rice hulls, grass, newspaper, corn cob) and showed a much higher pH (on day 0; 5.9–7.3, on day 35; 8.5–9) than in our research. According to the authors, a pH below 7 is an advantage, and the conversion of uric acid to ammonia is reduced at such a low pH.

As the pH rises above 7, ammonia becomes more volatile. The same conclusions were reported by Jie et al. [18]. At the same time, a too-low (acidic) pH may favor the growth of bacteria responsible for ammonia production [21]. Thus, it can be argued that the CHs in the litter positively affected the bedding quality due to the significantly higher pH than in the wheat straw pellet. Oliveira and Franca [5] described that CHs in the form of compost could be considered a soil-strengthening agent. It can have a physical effect on increasing water retention in soil and improving its long-term quality. Therefore, manure with CHs has the potential to use and manage waste.

Our research did not show the influence of pellet bedding with the share of CHs on the production results of broiler chickens. Monira et al. [27] found no differences in body weight, feed consumption rates, or survival of chickens reared on sawdust, rice husk, sugarcane bagasse, or wheat straw. Similarly, no effect of the type of bedding (sawdust, sand, and wheat straw) was shown by Hafeez et al. [28]. Only in the 4^th^ week of rearing (own research) a higher growth rate was demonstrated in the group of chickens kept on pellet bedding with a 50% share of CHs than in the group where the pellet bedding contained 10% of CHs. Researchers demonstrated better FCR in 10-day-old chickens on pellet bedding than those kept on rice husks [15]. In our and Kheravii et al. [15] research, no influence of the type of bedding on growth performance in the whole rearing period was found. Slight differences could be dependent on the condition of the bedding and its consumption by the birds. The differences in the growth rate of chickens may have been due to the moisture content of the bedding in the mentioned period. High-moisture bedding decreased the growth rate [29]. The growth of broilers may be adversely affected by an increase in the caking rate of bedding, where clumping reduces movements and the correct position of the chickens, thus presenting an inappropriate posture for feeding [14].

The environment in which chickens are kept can impact the birds’ development and meat quality characteristics. It is related to the possibility of exposing the birds to stress factors [30]. Oke et al. [11] showed a significant effect of bedding material on the weight of thigh muscles and intestines. Our study showed differences in carcass composition, which may result from significance values in pre-slaughter body and carcass weights of chickens selected for slaughter. The percentage of individual elements (especially muscles) was not significantly different. Costa et al. [30] did not show the effect of the used bedding (rice straw or shavings) on the quality characteristics of Cobb 500 chicken meat.

Similarly, Haung et al. [31] investigated the effect of bedding types (rice hulls, wood shavings, and coconut hulls) on the efficiency of Ross broiler production and showed no significant impact on the relative weight of the abdominal fat. In contrast, the other authors found a higher abdominal fat share from birds kept on the wood shavings than on the sand bedding [32]. During the evisceration of the carcasses, some of the abdominal fat deposited is lost, which can result in differences in results [33]. However, in our research, despite significant differences in the share of abdominal fat, the slaughter yield in all groups was maintained at a similar level. The differences between individual studies could be due to the different types of bedding, housing conditions, and even the genotype of birds.

Our research has shown a significant effect of adding CHs to straw-based pellets on the presence of footpad dermatitis (FPD). Sirri et al. [34] found less FPD in chickens on wood-shaving bedding than chopped straw. Yamak et al. [35] investigated the possibility of reusing bedding in the rearing of broiler chickens. Compared to the fresh bedding, an increase in FPD, caking, and manure scores in the re-used bedding was noticed. Cengiz et al. [29] analyzed the presence of FPD depending on the quality of the waste (unwetted or wetted, fresh pine shavings, either as-is or screened to a particle size of > 0.5 cm or < 0.5 cm, and used bedding with a particle size of < 0.5 cm). The authors concluded that early exposure to wet bedding could increase the frequency and severity of FPD, and the large particle size of the bedding has a direct and negative effect on the development of FPD. Skin changes on the chickens' foot pads depend on the moisture content of the bedding and its structure [36]. If the 25% and 50% coffee husk bedding was drier and did not form and clump, the CHs had a beneficial effect on the bedding characteristics and, thus, a reduced FPD level. Hunter et al. [37] state that litter moisture is related to FPD. The authors showed that using pine shavings bedding, litter depths of up to 12.7 cm, and dry bedding was associated with improved foot pad quality. Organic matter appears on the litter during rearing, including manure and feed remains. It can promote the growth of microorganisms and modify the bedding's moisture content [10]. It could have a potential impact on our results. However, feed effects must be ruled out as all chickens were fed similarly. Suppose the bedding quality affects the presence of FPD, which indicates the comfort of birds and their performance ability. In that case, the quality of meat and significant differences in our research could be caused indirectly by the research factor (different levels of stress feeling).

## Conclusions

In conclusion, this study showed that the pelleted bedding material, using coffee husks (CHs), strongly determines the chemical composition of the pellets. Along with the increase in the share of CHs in the pellet, a higher pH, nitrogen, and potassium content was demonstrated on day 42. The chemical composition of the pellets changed each week, which the chickens' excretions could also influence. The pelleted bedding with 25% and 50% CHs significantly reduced the incidence of skin lesions, leading to footpad dermatitis in broiler chickens. It could be related to bedding dry matter on day 42. When the highest dry matter was found, the lowest FPD was noticed. CHs did not have a negative effect on the performance characteristics of chickens and lower fatness of carcasses. The possibility of using CHs as a component in pellet bedding material is suggested. Reusing CHs could reduce waste disposal from the coffee industry, which aligns with pro-environmental trends (zero waste), especially since worldwide coffee production (including local coffee roasters) is still growing. Further research on the relationship between the use of CHs and the health status and incidence of footpad dermatitis in chickens should be continued due to the limited literature.

## Methods

### Animal ethics

The study was conducted on a farm cooperated with the Department of Animal Breeding and Nutrition, Bydgoszcz University of Science and Technology, Poland, granted by the National Science Center, Poland (project No. 2021/05/X/NZ9/00820). The study aimed to investigate effects of the use of coffee husks in pellet bedding on broiler chickens' performance characteristics and welfare indicators. All methods and procedures used in the study were approved by the authors’ Institutional Animal Care and Use Committee (Faculty of Animal Breeding and Biology, Bydgoszcz University of Science and Technology, Poland; Approval No. 2/2022). All methods followed relevant institutional guidelines, the ARRIVE guidelines, and the national legislation.

### Animals and experimental groups

A total of 200 Ross 308 broilers, with a mean initial weight of 38.26 g, were used in the experiment. One-day-old broilers were bought from the Hatchery House (Drobex-Agro Ltd., Solec Kujawski, Poland). The birds were kept for 42 days of rearing. On day 1, the chickens were divided into four equal groups. The birds within the groups were split into five repetitions (10 birds per pen). The pens were made of stainless steel mesh with a usable area of ​​1.5 m^2^. The birds were kept in the conditions of a small-scale farm. From day 1, the birds were provided with a room temperature of 32 °C (additional heaters were provided). In the following days, the temperature gradually decreased to 20 °C. Twenty-four hours of light were provided for the first three days. Then, a light cycle of 18 h and 6 h dark. From the 39^th^ day, the lighting time was extended to 23 h. The humidity in the room was 60–65%. The keeping of the birds was carried out according to the standard recommendations for rearing broiler chickens. Each pen had a bell-shaped drinker and a feeder (placed on the pen’s walls). Feed and water were given ad libitum. The commercial diet was purchased from a feed company (Golpasz, De Heus, Golub-Dobrzyń, Poland). As the company declared, the feed was balanced regarding nutrients and dedicated to broiler chickens. Three feeding phases were used: starter feed (1–14 days), grower feed (15–35 days), and finisher feed (36–42 days).

**Fig. 1:**
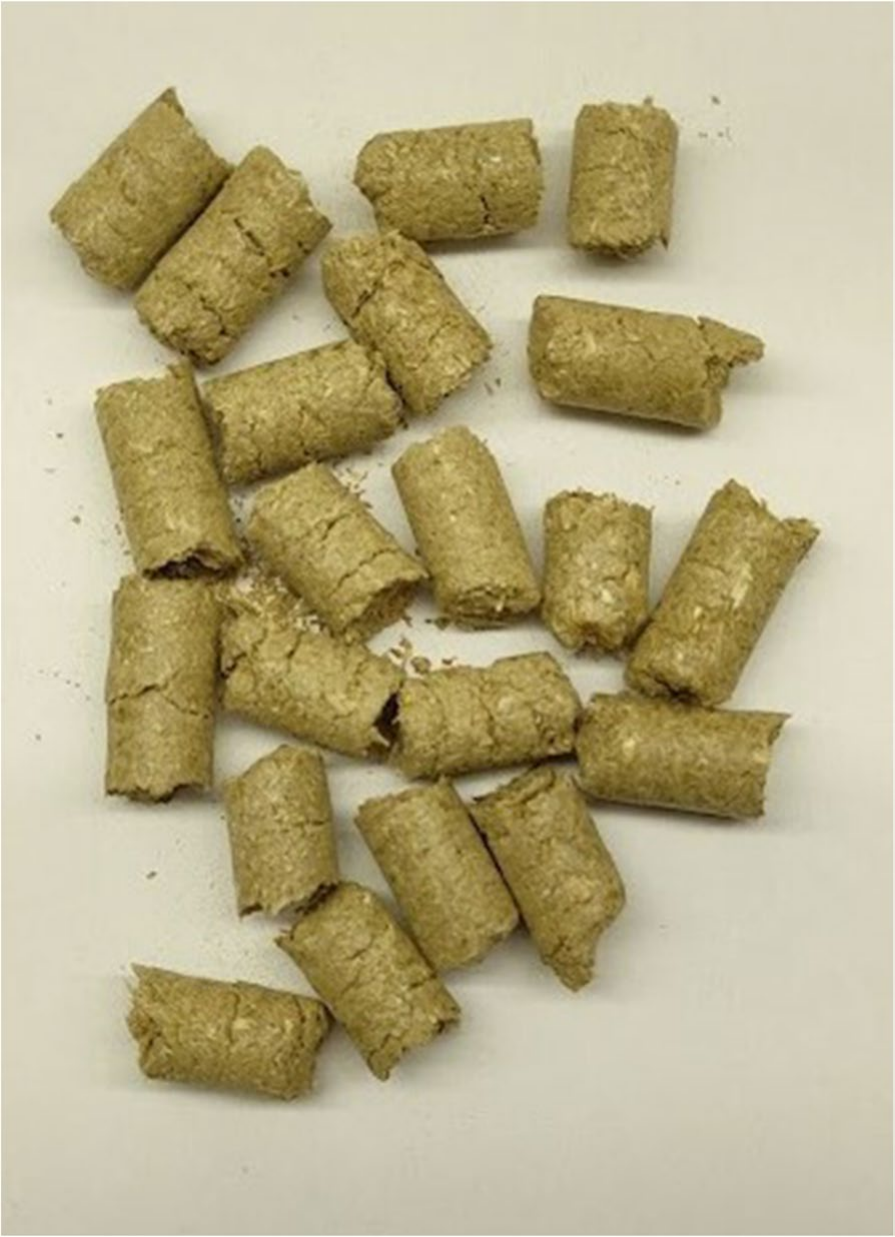
Pellet bedding based on wheat straw (control group)

**Fig. 2:**
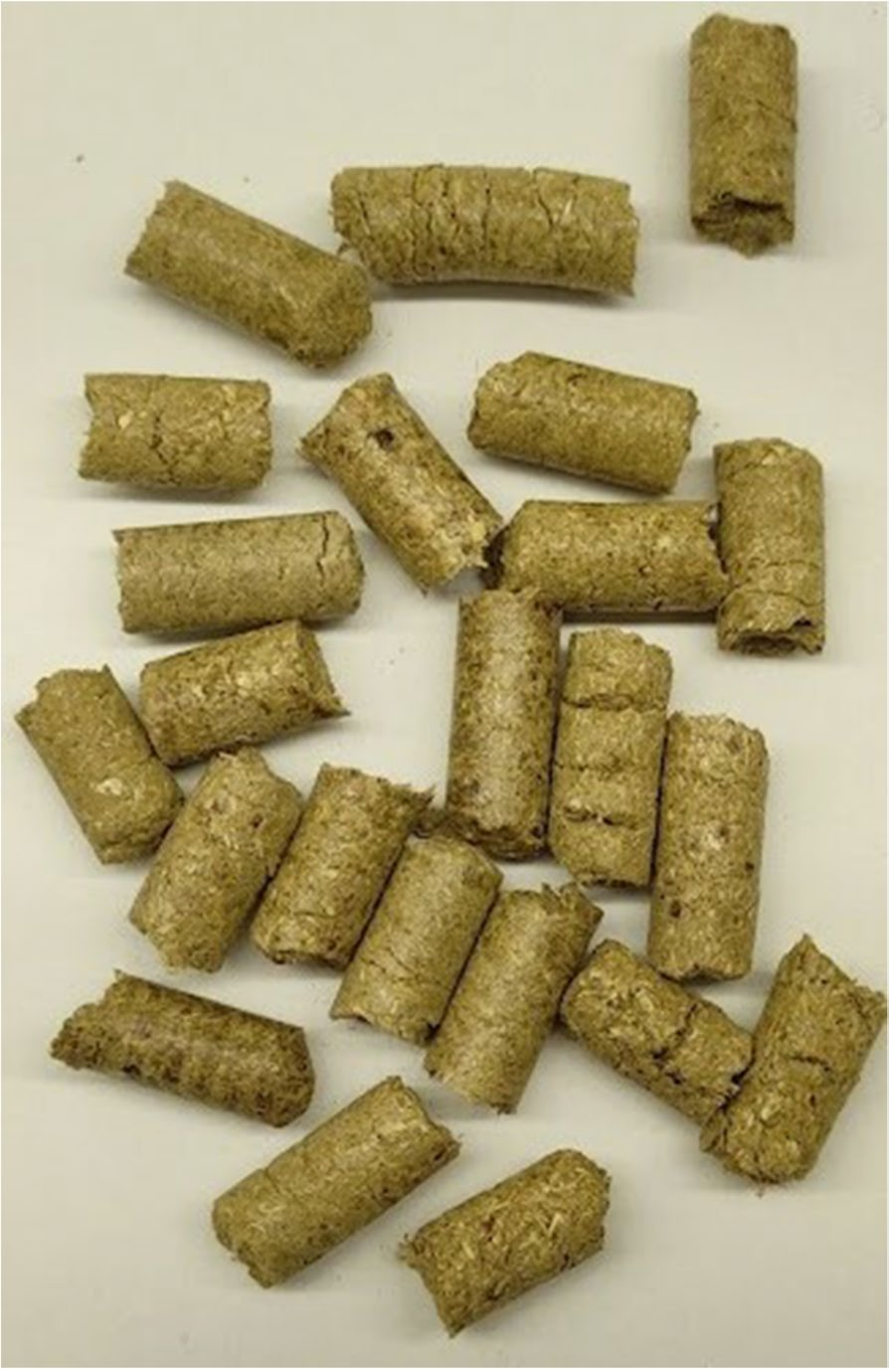
Pellet bedding based on 90% of wheat straw and 10% of coffee husks

**Fig. 3:**
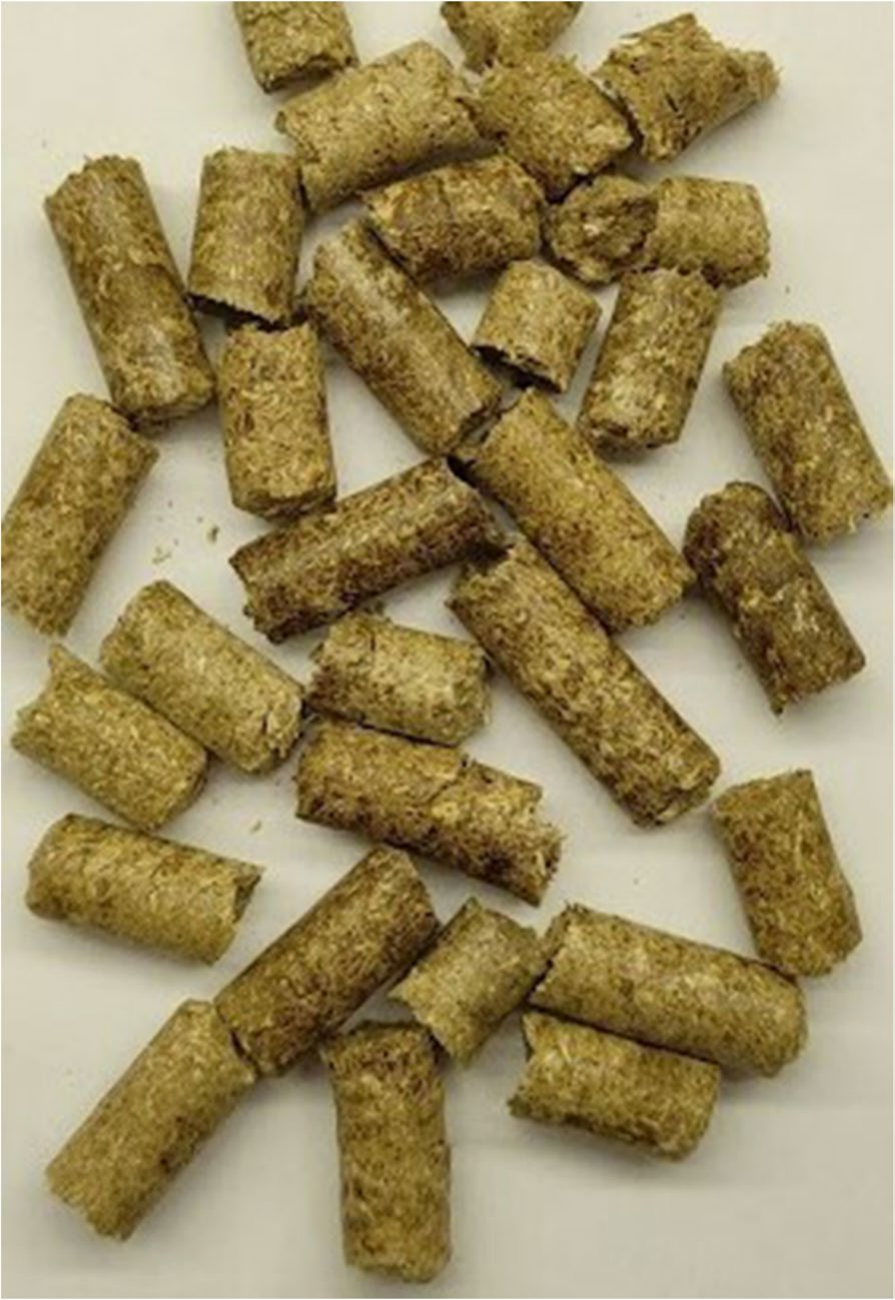
Pellet bedding based on 75% of wheat straw and 25% of coffee husks

**Fig. 4:**
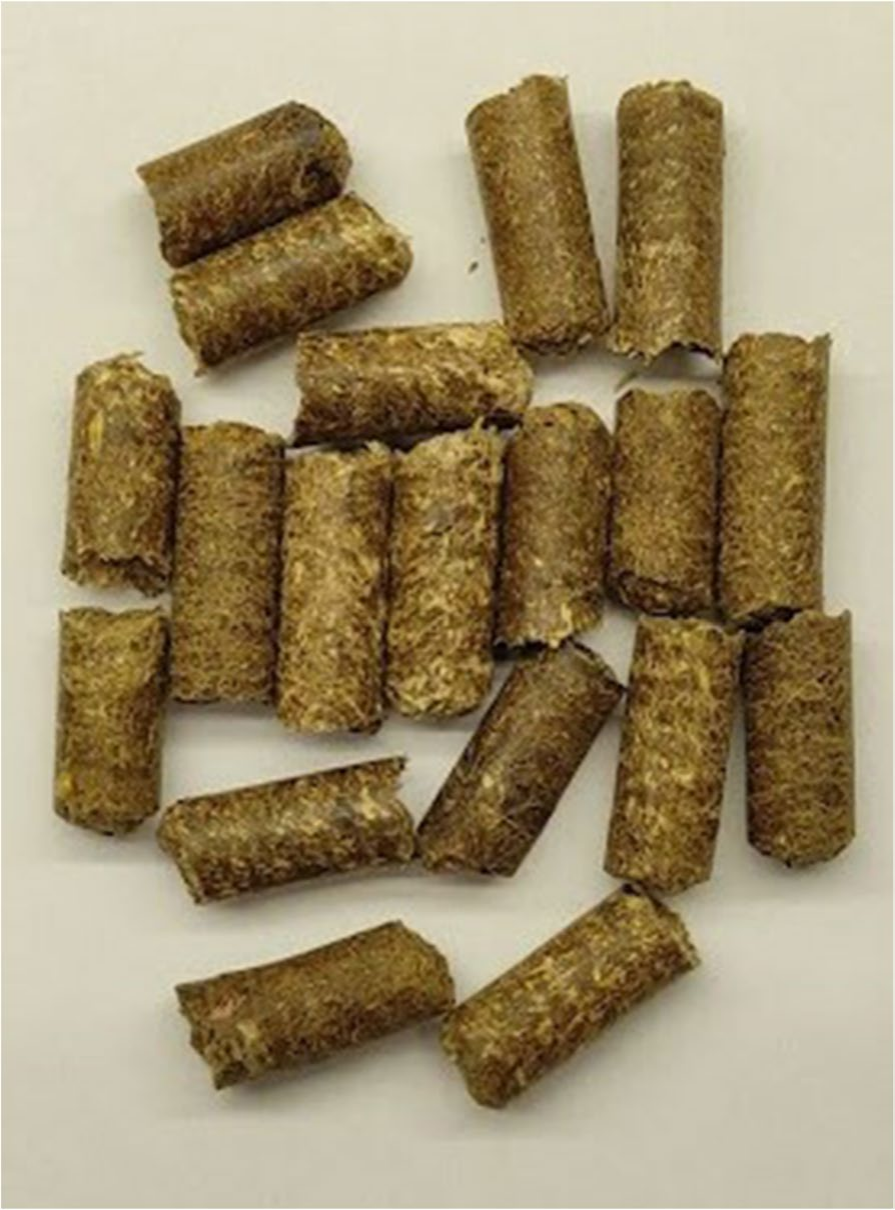
Pellet bedding based on 50% of wheat straw and 50% of coffee husks

The control group (C) was kept on a wheat straw pellet (100%) (Fig. 1). The experimental groups were kept on a pellet made of a mixture of wheat straw and CHs: group CH10, 90% wheat straw and 10% CHs (Fig. 2); CH25 group, 75% wheat straw and 25% CHs (Fig. 3); CH50, 50% wheat straw and 50% CHs (Fig. 4). The pellets were prepared using a granulator (RTH-150 pellet mill) (Pelleto, Poznań, Poland). The CHs were obtained from local coffee roasters in the Kuyavian-Pomeranian Voivodeship in Poland. To each pen, 6 kg of the pellet was added one day before the rearing started. The pellet preparation was carried out under patent application pending procedure number P.44383.

### Features of bedding

Pellet bedding was sampled from each pen on days 1, 7, 14, 21, 28, 35, and 42. The samples were collected in string bags. Physicochemical analyses were performed. The dry matter was determined based on the PN-ISO 6496:2002 method [38]. The crude fiber was determined based on the PN-ISO 6865:2002 method [39]. In turn, acid detergent fiber (ADF) and neutral detergent fiber (NDF) were done based on the PN-EN ISO 13906:2009 [40] and PN-EN ISO 16472:2007 [41] standards. The analysis used the gravimetric method. The Kjeldahl method also determined the nitrogen content. Phosphorus was determined by the photometric method. Potassium was analyzed by atomic absorption spectrometry (ASA) [42]. The pH value was analyzed by potentiometry. The tests were performed in 10 replications for each group (5 pens of 2 repetitions). The dry matter, crude fiber content, and pH value of raw CHs were also analyzed.

The firmness and toughness of pellets were done using the texture analyzer TA.XT plus C (Stable Micro System, Surrey, UK). A flat knife cut each piece of the pellet. The test speed was 1.50 mm/sec. The pellet was placed on a heavy-duty platform. The pellets’ absorptive capacity was based on the method of Bilgili et al. [43]. 20-g samples from each fresh bedding material were placed in nylon socks and fully submerged in water for 24 and 48 h. At the end of each interval, the socks were hung, gently massaged to drip, allowed to air-dry for 30 min, and subsequently reweighed. The percentage ratio of the sample weight to the initial weight was calculated.

After rearing, the bedding was assessed on the modified scale from the Welfare Quality Assessment Protocol (Welfare Quality® Consortium, 2009) described by Hunter et al. [37]. Bedding moisture was assessed on a 5-point scale. Hand sampling was done. The ten samples per group were collected in pens (near walls and between the feeder and the drinker). The scoring was as follows: 0, dry bedding moves easily when touched in hand; 1, bedding moves less easily in hand but does not form a ball; 2, bedding forms a ball when compressed, but it easily falls apart; 3, bedding stays in a ball when compressed, wet bedding; 4, bedding stays in a ball when compressed after the compacted surface crust is broken.

### Growth performance

The chickens were weighed (BW) during rearing, and the daily feed intake (FI) was recorded. Based on the obtained results, average daily body weight gain (ADBWG) = $\frac{BWG\;(g)}{amount\;of\;days}$, average daily feed intake (ADFI) = $\frac{FI\;(g)}{amount\;of\;days}$, feed conversion ratio (FCR) = $\frac{FI (g)}{BWG (g)}$. Deaths of chickens were also recorded. The EPEF (European Production Efficiency Factor) using the formula $(\frac{viability\;\left(\%\right) \times BW\;\left(g\right)}{42\;days \times FCR\;\left(\frac{kg}{kg}\right)})\div 10$ and EBI (European Broiler Index) using the formula $(\frac{viability \left(\%\right) \times ADBWG(g)}{FCR (\frac{kg}{kg})})\div 10$ were calculated.

### Slaughter yield and meat quality

On the 42^nd^ day of rearing, the chickens were weighed. Then, two birds from each replicate (*n* = 10 in each group) were selected for slaughter. The birds were stunned using an electric current in a water bath (applicable act: Council Regulation (EC) No 1099/2009 of 24 September 2009 on the protection of animals at the time of killing). Then, decapitation was performed by cutting off the head between the occipital condyle and the first cervical vertebra. There was rapid bleeding of the carcass. Carcasses were plucked in a mechanical plucker and eviscerated. The legs were severed at the hock. The eviscerated carcasses and edible offal (heart, liver, and gizzard) were chilled for 24 h in a refrigerator (Hendi, Poznań, Poland) at 4 °C.

On the next day, the carcasses and offal were weighed. Using a pH meter (Elmetron, Zabrze, Poland) equipped with a dagger electrode, the acidification of the muscle tissue in the pectoral muscles was measured (pH_24hours_). The pH meter was calibrated using known pH buffers: 4.00, 7.00, and 9.00. Then, a dissection was performed [44]. The neck (without skin), pectoral muscles (including pectoralis major and minor), leg muscles (thighs and shins, without bones), skin with subcutaneous fat (with skin from the neck), and fat and wings with skin were distinguished. The carcass' remains were the trunk and leg bones (femur and tibia).

The slaughter yield of the carcass (with or without offal) was calculated $(\frac{carcass\;weight\;\left(g\right) \left(with\;or\;without\;offal\right)}{pre-slaughter\;weight\;\left(g\right)}\times 100\%)$. Total muscle weight (sum of pectoral muscles and leg muscles) and fatness (sum of skin with subcutaneous fat and abdominal fat) were calculated. The percentage of all items in the carcass was calculated $(\frac{carcass\;{\prime} element\;weight\;\left(g\right)}{carcass\;weight\;\left(g\right)}\times 100\%)$. The pectoral muscles (left and right) and the leg muscles (left and right) were assigned for further qualitative analysis. Each chicken's right femur and tibia were used for breaking strength analysis.

The right pectoral and leg muscles were analyzed for color on the outer side. A colorimeter (Konica Minolta, Tokyo, Japan) was used for the tests. The method was performed on the CIE Lab-scale (Commission Internationale de I'Eclairage) [45]. The colorimeter was calibrated using a white calibration plate. Lightness (L*), redness (a*), and yellowness (b*) of the muscles were determined. Based on Honikiel's [46] method, drip loss of the pectoral (right) muscles was performed. The muscles were weighed (initial weight), placed in zip bags (the inner bag was partially cut in the lower part), and suspended in a 4 °C refrigerator for 24 h. The muscles were then reweighed (final weight). The percentage of water loss was calculated. The pectoral and leg muscles (left) were ground into a meat grinder within the groups. The minced meat was intended for water-holding capacity (WHC) [47]. The samples were weighed (initial weight). The initial sample weight was 0.300 g (± 0.005 g). The samples were placed between two pieces of Whatman 1 blotting paper, covered with a 2 kg load for 5 min, and weighed again (final weight). Similar to the drip loss analysis, the percentage of water loss was calculated. A formula $100-\left(\frac{final\;weight\;\left(g\right)}{initial\;weight\;\left(g\right)}\right)\times 100\%$ was used.

Analysis of bone-breaking strength (right femur and tibia) was performed using the Instron 3345 apparatus (Instron, Buckinghamshire, UK). It was integrated with the Bluehill 3 software. Bone strength was analyzed using the Instron Bend Fixture 10 mm Anvil adapter at a speed of 250 mm/min. The bones were placed between the clamps. The maximum load and force at break (N) were measured. Meat qualitative and bone-breaking strength' analyses were performed in 10 replicates. All the described analyses were done in the research by Biesek et al. [48].

### Welfare indicators – footpad dermatitis, hock burns, and feather scoring

Footpad dermatitis (FPD) analysis was performed according to the method described by Sorin et al. [49]. Data are presented as the percentage of individual skin lesions on the soles of the feet (% of the herd). The assessments were made on the soles of the feet of chickens without soiling on the day of slaughter. One person made the assessment. FPD score was as follows: 0, indicated no or very small superficial changes, slight discoloration of the skin of the sole, and mild hyperkeratosis; 1, showed mild skin changes and discoloration, as well as superficial changes, dark warts, and hyperkeratosis; 2; indicated severe lesions, ulcerations, and crusts, as well as signs of hemorrhages and swollen soles of the paws.

Based on the collected data, the FDF score (points) was calculated using the formula: $\frac{\left[100\times \left(0\times FPD\;with\;a\;score\;of\;0+0.5 \times FPD\;with\;a\;score\;of\;1+2\times FPD\;with\;a\;score \right)\right]}{of\;2\;total\;number\;of\;FPD\;scored}$.

Feather scores, or the degree of coverage of feathers on the breasts, were recorded. Each bird was stroked by a keel with the hand from front to back, and the amount of flesh visible through the pressed feathers was arbitrarily scored on a three-point scale (1, no visible skin, full feather coverage; 2, relatively small area of skin exposed; 3, rather large skin exposure area). The hock burns were scored using a three-point scale (1, no burns; 2, moderate burns; 3, severe burns). The assessment was done according to Thomas et al. [50].

### Statistical calculation

The numerical data were calculated in a statistical program (Statistica 13.3, 2017, Statsoft, TIBCO, Kraków, Poland). Each feature’s mean values and the mean’s standard error of the mean (SEM) were calculated. The data were analyzed for the normal distribution and homogeneity of the groups (Shapiro–Wilk test, Levene test). Statistically significant differences were verified with Tukey's test, assuming a *p*-value < 0.05. The interaction was checked. Statistical models were used for one-way (*Y*_BM/RD_ = *µ* + C_BM_/D_RD_ + e_BM/RD_) and two-way (*Y*_BMRD_ = *µ* + C_BM_ + D_RD_ + CD_BMRD_ + e_BMRD_) analysis of variance, where Y_BM/RD_ = the dependent variable; µ, the overall mean; C_BM_, effect of bedding material (BM = C, CH10, CH25, CH50); D_RD_, effect of rearing days (RD = 1, 7, 14, 21, 28, 35, 42); CD_BMRD_, interaction between bedding material and rearing days; e_BMRD_, residual error). The adjusted coefficient of determination (adj. R^2^) was also calculated. In the Results section, the overall *P*-value for bedding chemical composition was presented, and for production results and carcass features – detailed *P*-value from Tukey’s test.

## Data Availability

All datasets supporting the conclusions of this article are included within the article.

## Abbreviations

ADBWG: Average daily body weight gain
ADF: Acid detergent fiber
ADFI: Average daily feed intake
Adj. R^2^: Adjustable coefficient of determination
BW: Body weight
CHs: Coffee husks
EBI: European Broiler Index
EPEF: European Production Efficiency Factor
FCR: Feed conversion ratio
FPD: Footpad dermatitis
NDF: Neutral detergent fiber
SEM: Standard error of the mean
WHC: Water-holding capacity

## References

[1] da Rodrigues Silva M, Sanchez Bragagnolo F, LajarimCarneiro R, de Oliveira Carvalho Pereira I, Aquino Ribeiro JA, Martins Rodrigues C, Jelley RE, Fedrizzi B, Soleo Funari C (2022). Metabolite characterization of fifteen by-products of the coffee production chain: from farm to factory. Food Chem.

[2] Munirwan RP, Taha MR, Taib AM, Munirwansyah M (2022). Shear strength improvement of clay soil stabilized by coffee Husk Ash. Appl Sci.

[3] Głowacka R, Górska A, Wirkowska-Wojdyła M (2018). Coffee silverskin – new and natural alternative to generating selected bioactive compounds. Zesz Probl Postęp Nauk Rol.

[4] Emma AF, Alangar S, Yadav AK (2022). Extraction and characterization of coffee husk biodiesel and investigation of its effect on performance, combustion, and emission characteristics in a diesel engine. Energy Convers Manag: X.

[5] Oliveira LS, Franca AS. Chapter 31 – An overview of the potential used for Coffee Husks. In Coffee in Health and Disease Prevention. Ed. V.R. Preedy, ISBN: 978–0–12–409517–5, Academic Press, Elsevier Inc. 2015. p. 283 – 291. 10.1016/B978-0-12-409517-5.00031-0.

[6] Tolessa B, Tibba GS, Singh B (2022). Utilization of coffee husk as an alternative source: a current trend. Tierarztl Prax.

[7] Velusamy S, Subbaiyan A, Murugesan SR, Shanmugamoorthy M, Sivakumar V, Velusamy P, Veerasamy S, Mani K, Sundararaj P, Periyasamy S (2022). Comparative analysis of agro waste material solid biomass briquette for environmental sustaiability. Adv Mater Sci Eng.

[8] Sabogal-Otálora AM, Palomo-Hernández LF, Piñeros-Castro Y (2022). Sugar production from husk coffee using combined pretreatments. Chem Eng Process Process Intesification.

[9] Dzung NA, Dzung TT, Khanh VTP (2013). Evaluation of Coffee Husk Compost for Improving Soil Fertility and Sustainable Coffee Production in Rural Central Highland of Vietnam. Resour Envrion.

[10] de Souza CF, dos Santos CR, Inoue KRA, de Tinôco IFF, Ferreira WPM (2018). Additives to control the quality of coffee husk poultry litter. Rev Eng Agric.

[11] Oke OE, Daramola JO, Uyanga V, Iyasere OS, Njoku CP, Babatunde MB (2019). Influence of bedding materials on organ weights, meat quality, breast and footpad dermatitis of broiler chickens under hot humid climate. Agric Tropica Et Subtopica.

[12] Ortiz A, Valdivie M, Elias A (2003). Coffee husk as poultry bedding First rearing. Cuban J Agric Sci.

[13] Ortiz A, Valdivie M, Elias A (2003). Reuse of coffee husk as bedding on a second rearing of broilers. Cuban J Agric Sci.

[14] Farghly MFA, Mahrose KhM, Cooper RG, Metwally KhA, Abougabal MSh, El-Ratel IT (2021). Use of available crop by-products as alternative bedding materials to wheat straw for rearing broilers. Animal.

[15] Kheravii SK, Swick RA, Choct M, Wu S-B (2017). Potential of pelleted wheat straw as an alternative bedding material for broilers. Poult Sci.

[16] Kheravii SK, Swick RA, Choct M, Wu S. The impact of bedding materials on broiler performance. 26^th^ Ann. Aust. Poult. Sci. Symp. Sydney, Australia, 9^th^ – 11^th^ February 2015. p. 213. https://hdl.handle.net/1959.11/17520.

[17] Liao S-C, Lu P-X, Shen S-Y, Hsiao C-C, Lien C-Y, Wang S-D, Lin T-Y, Tu P-A (2021). Effects of different swimming pool conditions and floor types on growth performance and footpad dermatitis in INDOOR-Reared white roman geese. Animals.

[18] Jie D, Zhang Z, He J, Zhou Y, Zhu G (2022). Impact of waste tea litter on NH_3_ and CO_2_ emissions during broiler rearing. Appl Sci.

[19] Zapata OL, de Ferreira IF, Osorio JA, de Souza CF, de Araujo MF (2015). Evaluation of the fertilizer and contamination potential of different broiler litter types subjected to various use cycles. Rev Fac Nal Agr.

[20] Eser H, Onbasilar EE, Yalcin S, Ramay MS, Karakan T, Gunor OF, Yalcin S (2022). Comparison of litter quality, performance, and some welfare parameters of broilers reared on the sepiolite-supplemented paper waste sludge. Environ Sci Pollut R.

[21] Živkov Baloš M, Knežević S, Pajić M, Popov N, Jakšić S, Vidaković Knežević S, Mihaljev Ž, Bugarski D (2020). The effects of bedding material containing peat moss on broiler production performance and fertilizing quality of the litter. Arhiv Vet Med.

[22] Robinson JS, Sharpley AN (1995). Release of nitrogen and phosphorus from poultry litter. J Environ Qual.

[23] Font-Palma C (2012). Characterisation, kinetics and modelling of gasification of poultry manure and litter: an overview. Energy Convers Manag.

[24] Knudsen KEB (2014). Fiber and nonstarch polysaccharide content and variation in common crops used in broiler diets. Poult Sci.

[25] Petric I, Šestan A, Šestan I (2009). Influence of wheat straw addition on composting of poultry manure. Process Saf Environ Prot.

[26] Garces A, Afonso SMS, Chilundo A, Jaiorce CTS (2013). Evaluation of different litter materials for broiler production in a hot and humid environment: 1 Litter characteristics and quality. J Appl Poult Res.

[27] Monira KN, Islam MA, Alam MJ, Wahid MA (2003). Effect of litter materials on broiler performance and evaluation on manureal value of used litter in late autumn. Asian Australas J Anim Sci.

[28] Hafeez A, Suhail S, Durrani F, Jan D, Ahmed I, Rehman A (2009). Effect of different types of locally available litter materials on the performance of broiler chicks. Sarhad J Agric.

[29] Cengiz Ö, Hass JB, Bilgili SF (2011). Effect of bedding type and transient wetness on footpad dermatitis in broiler chickens. J Appl Poult Res.

[30] Costa HDA, Vaz RGMV, Silva MCD, Rodrigues KF, Sousa LF, Bezerra LDS, Ribeiro MDC, Barbosa AFC, Almeida JSD, Oliveira MFD (2021). Performance and meat quality of broiler chickens reared on two different litter materials and at two stocking densities. Brit Poult Sci.

[31] Haung Y, Yoo JS, Kim HJ, Wang Y, Chen YJ, Cho JH, Kim IH (2009). Effect of bedding types and different nutrient densities on growth performance, visceral organ weight, and blood characteristics in broiler chickens. J Appl Poult Res.

[32] de Souza LFA, Massaranduba NT, de Almeida RI, de Souza A, Gomes APSC, Silva AFG (2016). Performance, carcass yield and behavior of broilers reared on wood shavings or sand bed. Colloquium Agrariae.

[33] Health JL, Covery RC, Owens SL (1980). Abdominal leaf fat separation as a result of evisceration of broiler carcasses. Poult Sci.

[34] Sirri F, Minelli G, Folegatti E, Lolli S, Meluzzi A (2007). Foot dermatitis and productive traits in broiler chickens kept with different stocking densities, litter types and light regimen. Ital J Anim Sci.

[35] Yamak US, Sarica M, Boz MA, Uçar A (2016). Effect of reusing litter on broiler performance, footpad dermatitis and litter quality in chickens with different growth rates. Kafkas Univ Veteriner Fakultesi Dergisi.

[36] Zikic D, Djukic-Stojcic M, Bjedov S, Peric L, Stojanovic S, Uscebrka G (2017). Effect of litter on development and severity of footpad dermatitis and behavior of broiler chickens. Braz J Poult Sci.

[37] Hunter JM, Anders SA, Crowe T, Korver DR, Bench CJ (2017). Practical assessment and management of foot pad dermatitis in commercial broiler chickens: a field study. J Appl Poult Res.

[38] PN-ISO 6496:2002. Pasze – oznaczanie wilgotności i zawartości innych substancji lotnych. 2002. (in Polish). https://www.pkn.pl/.

[39] PN-ISO 6865:2002. Pasze – oznaczanie zawartości włókna surowego – metoda z pośrednią filtracją. 2002. (in Polish). https://www.pkn.pl/.

[40] PN-EN ISO 13906:2009. Pasze – oznaczanie zawartości włókna kwaśnodetergentowego (ADF) i ligniny kwaśnodetergentowej (ADL). 2009. (in Polish). https://www.pkn.pl/.

[41] PN-EN ISO 16472:2007. Pasze – oznaczanie zawartości włókna obojętnodetergentowego po traktowaniu amylazą (aNDF). 2007. (in Polish). https://www.pkn.pl/.

[42] PN-EN ISO 6869:2002. Pasze – oznaczanie zawartości wapnia, miedzi, żelaza, magnezu, manganu, potasu, sodu i cynku – metoda absorpcyjnej spektrometrii atomowej. 2002. (in Polish). https://www.pkn.pl/.

[43] Bilgili SF, Hess JB, Blake JP, Macklin KS, Saenmahayak B, Sibley JL (2009). Influence of bedding material on footpad dermatitis in broiler chickens. J Appl Poult Res.

[44] Ziołecki J, Doruchowski W (1989). Methods for Assessing Slaughter Value.

[45] CIE. Colorimetry. Publication CIE 15.2. Central Bureau of CIE: Vienna, Austria; 1986.

[46] Honikel KO (1987). The water binding of meat. Fleischwirtschaft.

[47] Grau R, Hamm R (1952). Eine einfache methode zur bestimmung der wasserbindung in fleisch. Fleischwirtschaft.

[48] Biesek J, Banaszak M, Wlaźlak S, Adamski M (2022). The effect of partial replacement of milled finisher feed with wheat grains on the production efficiency and meat quality in broiler chickens. Poult Sci.

[49] Sorin MC, Ilie V, Georgeta C, Anca G, Teodor M (2013). Influence of the dietary protein level on the incidence of footpad dermatitis in broiler chickens. Indian J Anim Sci.

[50] Thomas DG, Ravindran V, Thomas DV, Camden BJ, Cottam YH, Morel PCH, Cook CJ (2004). Influence of stocking density on the performance, carcass characteristics, and selected welfare indicators of broiler chickens. New Zealand Vet J.

